# Hotel Dieu

**Published:** 1831-01

**Authors:** 


### HOSPITAL REPORTS.

### HOTEL DIEU.

### *Phlebitis of the Saphena Veins cured by Compression.**

May 27, 1830.—A young man, æt. seventeen, was admitted, who had been obliged to remain constantly in a standing posture. He was of a feeble habit, and complained of pain and debility of the lower extremities. He had had diarrhœa for two days. This symptom soon disappeared, but the pains in the legs increased. Baths were employed without advantage. The continuance of pain led to a more attentive investigation of the case; and on the 7th of June, upon examining the lower extremities, small and irregular enlargements, of a blueish colour, were detected upon the course of both internal saphena veins. These enlargements were very painful on pressure, and did not disappear under the weight of the finger. The inguinal glands were not tumefied. There were no red streaks of the skin, indicative of any affection of the lymphatic system. The patient could not bend his knees without pain, on account of

* Journal Complémentaire.

a slight swelling in the ham, which was supposed to depend upon enlargement of the glands of the part, from obstruction to the venous circulation. The pain did not run in the track of the nerves, and was not, indeed, present except when the swelling was pressed upon, when it became very acute. There was little or no fever.

The case was now considered to be partial phlebitis of both saphenæ veins, and M. Recamier prescribed free doses of tartar emetic, which caused vomiting. No benefit was derived from this treatment, which, however, was scarcely continued long enough to enable any opinion to be formed of its efficacy. Compression was determined upon. Firm poultices of linseed were applied, and bound down by rather tight bandages.

The following day, the tumors on the veins were diminished in size, and less pain was felt on pressure. There was an obvious amendment every day, and in a short time all traces of phlebitis had ceased. Compression was still, however, continued, as the pains in the legs had not entirely disappeared.

It is to be observed, that the pains continued some time after the tumors, which showed the character of the disease had diminished. The limbs at length recovered their strength and action, and the patient quitted the hospital entirely cured.

